# Translating shared decision-making into health care clinical practices: Proof of concepts

**DOI:** 10.1186/1748-5908-3-2

**Published:** 2008-01-14

**Authors:** France Légaré, Glyn Elwyn, Martin Fishbein, Pierre Frémont, Dominick Frosch, Marie-Pierre Gagnon, David A Kenny, Michel Labrecque, Dawn Stacey, Sylvie St-Jacques, Trudy van der Weijden

**Affiliations:** 1 Centre hospitalier universitaire de Québec, Hôpital St-François D'Assise, Unité de recherche évaluative, 10 rue de l'Espinay, Québec, Québec, G1L 3L5, Canada; 2 Department of Primary Care and Public Health, School of Medicine, Cardiff University, Neuadd Meirionnydd, Heath Park CF 14 4YS, UK; 3 Annenberg School for Communication, University of Pennsylvania, 3620 Walnut Street, Philadelphia, PA 19104, USA; 4 UCLA Med-GIM & HSR, BOX 951736, 911 Broxton, Los Angeles, CA 90095-1736, USA; 5 Department of Psychology, University of Connecticut, 406 Babbidge Road Unit 1020 Storrs, CT 06269-1020, USA; 6 School of Nursing, University of Ottawa, 451 Smyth, Room RGN 3247A Ottawa, ON K1H 8M5, Canada; 7 Department of General Practice/School of Public Health and Primary Care Caphri, Maastricht University, PO Box 616, 6200 MD Maastricht, The Netherlands

## Abstract

**Background:**

There is considerable interest today in shared decision-making (SDM), defined as a decision-making process jointly shared by patients and their health care provider. However, the data show that SDM has not been broadly adopted yet. Consequently, the main goal of this proposal is to bring together the resources and the expertise needed to develop an interdisciplinary and international research team on the implementation of SDM in clinical practice using a theory-based dyadic perspective.

**Methods:**

Participants include researchers from Canada, US, UK, and Netherlands, representing medicine, nursing, psychology, community health and epidemiology. In order to develop a collaborative research network that takes advantage of the expertise of the team members, the following research activities are planned: 1) establish networking and on-going communication through internet-based forum, conference calls, and a bi-weekly e-bulletin; 2) hold a two-day workshop with two key experts (one in theoretical underpinnings of behavioral change, and a second in dyadic data analysis), and invite all investigators to present their views on the challenges related to the implementation of SDM in clinical practices; 3) conduct a secondary analyses of existing dyadic datasets to ensure that discussion among team members is grounded in empirical data; 4) build capacity with involvement of graduate students in the workshop and online forum; and 5) elaborate a position paper and an international multi-site study protocol.

**Discussion:**

This study protocol aims to inform researchers, educators, and clinicians interested in improving their understanding of effective strategies to implement shared decision-making in clinical practice using a theory-based dyadic perspective.

## Background

With the increased emphasis on engagement of patients as partners in their care, there is a need to determine effective ways to involve patients in the process by which health-related decisions are made in clinical settings. The health decision-making process is complex, as it brings together a health professional, considered a scientific content expert, and an individual, considered an expert in his own personal values [1]. It is in this context that there is considerable interest today in the process of shared decision-making (SDM) [2]. SDM is defined as a decision-making process jointly shared by patients and their health care provider [3], and is said to be the crux of patient-centered care [4]. It relies on the best evidence about risks and benefits associated with all available options (including doing nothing) and on the values and preferences of patients, without excluding those of health professionals [5]. Therefore, it includes the following components: establishing a context in which patients' views about treatment options are valued and deemed necessary; reviewing the patient's preferences for role in decision-making; transferring technical information; making sure patients understand this information; helping patients base their preference on the best evidence; eliciting patients' preferences; sharing treatment recommendations; and making explicit the component of uncertainty in the clinical decision-making process [6]. However, a recent systematic review identified 161 conceptual definitions of SDM, thus suggesting that SDM as a concept is still an object of ongoing research [7].

Patient decision aids and decision coaching are effective interventions to support patients to engage in SDM. When compared to usual care, decision aids reduce patients' passivity in the decision-making process, improve patients' knowledge about clinical options, increase realistic expectations, reduce decisional conflict and the number of individuals who remain undecided, increase satisfaction with the decision-making process, and increase congruence between patient preferences and clinical options selected [8]. Moreover, notwithstanding the preferred role of patients, active participation of patients in the decision-making process correlates with improved quality of life measured three years after the decision [9].

The data show that SDM has not been broadly adopted yet [10-13]. There are major barriers to overcome in the goal of diffusion or dissemination of new approaches in clinical practice [14,15]. In a systematic review of barriers and facilitators to implementing SDM and patient decision aids in clinical practice as perceived by health professionals [16], among 28 unique studies that had collected data from 15 countries, the three most often reported barriers were: time constraints, lack of applicability due to patient characteristics, and lack of applicability due to the clinical situation. These results suggest that health professionals might be selecting, *a priori*, certain patients for whom they believe that SDM is feasible or functional. This is of some concern because physicians may misjudge patients' desire for active involvement in decision-making [17]. These results highlight the importance of the patient's input for successful implementation of SDM and patient decision aids in clinical practice. Hence, the concomitant evaluation of patients' and providers' perception of the decision-making process (dyadic decision-making) remains unavoidable for those interested in a comprehensive understanding of clinical decision-making [18].

In recent years, social cognitive theoretical models have been used to improve our understanding of health care behaviors [19,20] and health care professionals' behaviors [21-23]. At the time this research protocol was proposed, most of the studies that had been conducted to improve our understanding of the implementation of SDM in clinical practice had no clear theoretical basis. This is of some concern because it has been acknowledged that more attention needs to be given to the combination of different theories that could help us understand professional behaviours [14,24] and design effective implementation strategies [25]. Nonetheless, when social cognitive theoretical models have been used to study health care-related behaviors, such as communication during a consultation or the patient's adherence to medical advice, groups of patients and groups of health professionals have been studied separately as if living in separate worlds. This is a source of concern because 'the right thing to do' may only emerge in the course of the professional's contact with patients or clients [26]. Considering simultaneously both perspectives of the decision-making process is a logical approach for conceptualizing SDM and its implementation in clinical practice, as well as for identifying which aspects should be jointly evaluated by patients and their providers [27].

However, the study of dyads poses specific conceptual as well as methodological issues [28], and thus several challenges in advancing knowledge in this area remain, including the lack of consensus on which aspects should be jointly evaluated by patients and their providers; the absence of standardized measures with established psychometric properties; and the failure to take into account the clustering of patients under health providers [29]. In the majority of the studies pertaining to the relationship between a patient and a health care provider, very few have adequately addressed these methodological issues. The expertise, analytical strategies, and theoretical frameworks for studying dyads that have emerged in relationship studies [28,30-32] have the potential to enhance the theoretical underpinnings and the research methods for studying the implementation process of SDM in clinical practice because many dyadic processes are at play: patient-health provider, patient-family member, and health provider-health provider, to name only a few.

Consequently, the main goal of this new international collaboration is to bring together the resources and the expertise needed to develop an interdisciplinary and international research team dedicated to the study of implementing SDM in clinical practice using a theory-based dyadic perspective. Its objectives are: 1) to develop a collaborative research network in this area; 2) to test new strategies to analyze dyadic data and explore the impact of such analysis on the theoretical underpinnings guiding the implementation of SDM in clinical practice; and 3) to define a research agenda and best practices regarding the implementation of SDM in clinical practice.

## Methods

### Participants

Participants include researchers from Canada, US, UK, and Netherlands representing medicine, nursing, psychology, community health, and epidemiology. Team members from Canada contribute to this project by: 1) coordinating the proposed international collaboration; 2) hosting the workshop; 3) providing the necessary monitoring and on-going support that is required for an international research group to evolve and develop; 4) hosting the internet-based forum and collating relevant material to be shared with the team members; 5) sharing their experience and expertise in the development of a dyadic approach to the implementation of SDM in clinical practice and the data management of large existing datasets; 6) offering a unique perspective to implementing SDM in nursing clinical practice [33,34]; and 7) providing datasets to be used during the workshop.

Team members from other countries contribute to this project by: 1) providing extensive expertise in SDM at both the conceptual and methodological levels [6,13,35-37] and in implementation sciences [38-42]; 2) sharing their experience in producing and conducting clinical trials evaluating patient decision aids [43] and implementation strategies [38-42]; and 3) providing datasets to be used during the workshop.

Other collaborators from the US are the two key invited presenters at the two-day workshop. Together, they will bring extensive expertise on the theoretical underpinnings of implementing behavioral change [44-46], the study of interpersonal influences [28] and the analysis of dyadic data [47].

### Research activities

In order to develop a collaborative research network that draws upon the extensive theoretical, methodological and implementation expertise as well as on the extensive clinical research background in SDM of the investigators involved in the project, we propose to:

#### 1) Foster ongoing communication among members of this international research network

At the outset of the project, using internet-based forum or conference calls hosted by the group at Université Laval, all participants discuss a similar definition of the problems and challenges with implementing SDM, including methodological issues with analysis of dyadic data. Participants share relevant literature within the group and start to think about how this applies to the identified problems/challenges. Relevant collated documents are used to create a knowledgebase that can be shared through a website. An e-journal club dedicated to the critical appraisal of relevant health-related dyadic studies is proposed. It is possible that other issues that are truly unique to SDM will be identified. Ongoing communication is encouraged through a bi-weekly e-bulletin that is sent to all participants.

#### 2) Provide a workshop

A two-day workshop in Quebec City will be based on the previous work and expertise of participants. Each participant will be asked to prepare a short presentation outlining how they propose to address the following three research questions: 1) What are the most appropriate theoretical frameworks to assess how health professionals and patients engage in SDM, and what are the most appropriate theoretical frameworks to guide implementation of SDM in clinical practice? 2) What are the most appropriate measures to assess how health professionals and patients concomitantly engage in SDM, and what is the impact of SDM on both? 3) What are the most appropriate strategies and frameworks to analyze dyadic data that are nested under health professionals?

#### 3) Perform secondary analyses of existing dyadic datasets

One of the purposes of the workshop is to use existing dyadic datasets to explore the research questions presented above. This will ensure that the team's discussions are grounded in data. A dyadic dataset is defined as a dataset that include data on both members of a dyad that is a pair of two individuals. When only one member of the dyad is measured, the design is termed one-sided. When both members are measured on the same variable, the design is termed two-sided or reciprocal. Three different types of dyadic designs can be identified: 1) standard dyadic design in which each individual is linked to one and only one other individual in the sample; 2) one-with-many design in which one individual is linked to many other individuals; and 3) Social Relation Model design in which each individual is paired with multiple others, and each of these others is also paired with multiple others [47]. In this project, secondary analyses of existing dyadic datasets with a reciprocal one-with-many design will be favoured.

##### Sources of data

Previous trials and ongoing pilot trials of SDM in primary care were selected because they include the same measures at both the practitioner and patient levels. FL will provide a data set of 122 primary care providers and their 923 patients [48], and a data set of about 15 family practitioners and 51 pregnant women facing a decision about prenatal testing (on-going study). FL and ML will provide a data set of 36 to 60 family practitioners and 450 to 750 patients facing a decision about the use of antibiotics in acute respiratory infections [49]. DF will provide a dataset of about eight general practitioners and 164 adults facing a decision about prostate cancer and colorectal screening (ongoing study).

##### Data collected and variables assessed

Two datasets have data based on the Integrative Model of Behaviour [50] including the following variables: intention, attitude, social norm, and self-efficacy regarding engaging in SDM from the perspective of both providers and patients. The two datasets will be pooled. Based on the Ottawa Decision Support Framework [51,52], three datasets have data from the Decisional Conflict Scale [53], which was administered to both providers and patients after a specific clinical encounter. Based on the existing literature, all constructs that will be used in the planned analyses have excellent psychometrics in both languages (French and English) in both providers and patients.

##### Data analysis

Existing datasets will be combined. Proper handling of missing data will be ensured and simple descriptive statistics will be computed. Diverse dyadic indexes will then be tested between constructs assessed both in patients and providers [47]. The Actor-Partner-Interdependence Model (APIM) will be used to assess concomitantly in patients and providers the relationship between constructs [31].

#### 4) Build capacity

When and where possible, graduate students of the co-investigators will be invited to join the think tank sessions, participate in the e-journal club using the internet-based forum, and attend the two-day workshop. If appropriate, graduate students will be invited to participate in data synthesis and hypothesis testing activities.

#### 5) Elaborate a position paper and an international multi-site study protocol

A position paper defining a research agenda and best practices regarding the implementation of SDM in clinical practice using a theory-based dyadic perspective will be published. The team will develop an international multi-site study protocol that is based on the work accomplished during this project. The overarching goal of this study is to support both health professionals and individuals to engage in SDM. Based on the strong record of research excellence of all co-investigators and on existing dyadic data sets to be analyzed during the workshop, our research team is firmly convinced that it will attract funding for future projects.

## Discussion

'Good theories determine what one can see and discover in nature. Cutting-edge research methods and statistical techniques can influence what scientists see and discover in their data but also inform and change the way in which scientists think theoretically'[47]. This study protocol aims to inform researchers, educators, policy makers, and clinicians interested in designing and/or conducting implementation studies of SDM in clinical practice using a theory-based dyadic perspective. Although some international collaboration has been initiated between some of the team members, there are currently no coordinated efforts to enhance the research capacity at the international level to create a knowledgebase for implementing SDM in clinical practice using a theory-based dyadic perspective. Also, to the best of our knowledge, the proposed project does not duplicate other current international research effort in the area of implementation of SDM in clinical practice using a theory-based as well as a dyadic perspective. Therefore, this international collaboration addresses the many challenges associated with the systematic failure of implementing change in clinical practice by ensuring that future implementation research will take into account that the health professional's position is one that is ultimately 'relationship-centered' [54], and thus needs to be appraised within a dyadic perspective.

The deliverables of this Canadian Institute of Health Research (CIHR) funded research initiative are many: International and interdisciplinary group of researchers dedicated to implementing SDM in clinical practice using a dyadic perspective; conceptual and analytical approaches that will be used in future implementation of SDM in clinical practice studies; secondary data analyses of existing dyadic datasets; capacity building; a position paper defining a research agenda and best practices regarding the implementation of SDM in clinical practice; and a protocol for an international multi-site study on the implementation of SDM clinical practice.

In line with four of the eleven priority research themes of the Institute of Health Services and Policy Research of the Canadian Institute of Health Research, these deliverables are important as they will: Provide innovative insight on how to successfully implement change in clinical practices using a theory-based dyadic perspective; be helpful for future research on new models of collaborative care within the workforce environment related to health care provider-patient dyads; serve as a strategy to increase quality of care and patient safety; and reinforce a patient-centered care approach, one that highly values relationships [55]. Lastly, this international research initiative is in line with research priorities on social interactions of the Canadian Institute for Advanced Research whose mission is to 'incubate ideas that go on to revolutionize the international research community, and change the lives of people all over the world.' In summary, the proposed initiative is of foremost importance since it fosters a critical mass of research activities within an international network on the implementation of SDM in clinical practice and highlights a new paradigm in implementation science by putting forward a theory-based dyadic perspective.

## Competing interests

The author(s) declare that they have no competing interests.

## Authors' contributions

All authors collectively drafted the research protocol and approved the final manuscript. FL is its guarantor.
